# Improving adherence to medication in stroke survivors (IAMSS): a randomised controlled trial: study protocol

**DOI:** 10.1186/1471-2377-10-15

**Published:** 2010-02-24

**Authors:** Ronan O'Carroll, Martin Dennis, Marie Johnston, Cathie Sudlow

**Affiliations:** 1 Department of Psychology, Stirling University, Stirling, UK; 2 Department of Clinical Neurosciences, Western General Hospital, Crewe Road, Edinburgh, UK; 3 Psychology Department, William Guild Building, University of Aberdeen, Aberdeen, UK

## Abstract

**Background:**

Adherence to therapies is a primary determinant of treatment success, yet the World Health Organisation estimate that only 50% of patients who suffer from chronic diseases adhere to treatment recommendations. In a previous project, we found that 30% of stroke patients reported sub-optimal medication adherence, and this was associated with younger age, greater cognitive impairment, lower perceptions of medication benefits and higher specific concerns about medication. We now wish to pilot a brief intervention aimed at (a) helping patients establish a better medication-taking routine, and (b) eliciting and modifying any erroneous beliefs regarding their medication and their stroke.

**Methods/Design:**

Thirty patients will be allocated to a brief intervention (2 sessions) and 30 to treatment as usual. The primary outcome will be adherence measured over 3 months using Medication Event Monitoring System (MEMS) pill containers which electronically record openings. Secondary outcomes will include self reported adherence and blood pressure.

**Discussion:**

This study shall also assess uptake/attrition, feasibility, ease of understanding and acceptability of this complex intervention.

**Trial Registration:**

Current Controlled Trials ISRCTN38274953

## Background

Adherence to therapies is a primary determinant of treatment success. Poor adherence attenuates optimum clinical benefits and therefore reduces the overall effectiveness of health systems, yet it is estimated that in developed countries, only 50% of patients who suffer from chronic diseases adhere to treatment recommendations [1]. In the treatment of hypertension, it has been estimated that only 30-50% of patients regularly take their antihypertensive drugs as prescribed, and that non-adherence may cause half of antihypertensive drug "failures" [2]. A recent illustrative example from the field of cardiovascular disease is provided by the Duke Databank for Cardiovascular Disease for the years 1995 to 2002, which assessed the annual prevalence and consistency of self-reported use of aspirin, β-blockers, lipid-lowering agents, and combinations of the 3 drugs in patients with coronary artery disease. Rates of consistent self-reported medication use were sobering: aspirin (71%), β-blocker (46%), lipid-lowering agent (44%), aspirin and β-blocker (36%), and 21% for all 3 medications. Overall, consistent use was associated with lower adjusted mortality, although in this study the authors were unable to differentiate patient non-adherence from physician non-prescription [3]. In a further study of drug adherence and mortality in 31,455 survivors of myocardial infarction who were taking statins and β-blockers, patients were divided into 3 adherence categories: high, intermediate and low. After 1 year, compared with the high-adherence group, low adherers to statin therapy had a 25% increased risk of mortality [4]. Thus, polypharmacy is the norm, self-reported adherence is often suboptimal, and this is associated with elevated mortality risk [5].

The most recent Cochrane review of interventions to improve medication adherence concluded that, "Current methods of improving adherence for chronic health problems are mostly complex and not very effective, so that the full benefits of treatment cannot be realized. High priority should be given to fundamental and applied research concerning innovations to assist patients to follow medication prescriptions for long-term medical disorders" [6]. Our aim is to improve medication adherence in the secondary prevention of stroke. Stroke is the third most common cause of death in the UK, and is the most common cause of severe physical disability amongst adults. The National Audit Office recently estimated that the annual cost of caring for people with stroke is £7 billion per year in the UK alone [7]. The risk of a recurrent stroke is 30-43% within 5 years, and it is estimated that, currently, 11,626 strokes occur annually in the Scottish population [8]. Large randomised controlled trials and meta-analyses have identified several drugs which significantly reduce the risk of future vascular events after stroke. The Scottish Intercollegiate Guidelines Network (SIGN) guidelines for secondary prevention after stroke now recommend antiplatelet therapy and reduction of both blood pressure and cholesterol level [9]. The estimated efficacy of these drugs in helping prevent a further stroke in Scotland is outlined in Table 1.

**Table 1:** Estimated impact of pharmaceutical interventions to prevent recurrent strokes in Scotland

Intervention	Number of Strokes avoided	% of all strokes in Scotland
Aspirin	926	(8%)

Statins to reduce cholesterol	854	(7%)

Drugs to reduce blood pressure	751	(6%)

Dipyridamole added to aspirin	432	(4%)

Anticoagulants	376	(3%)

Taken from Health in Scotland 2007, Annual Report of the Chief Medical Officer, based on Table 18.4 in Warlow et al. [8]. The numbers in the table are derived from applying estimates from randomised trials and meta-analyses of the reduction in stroke risk achieved by adding each intervention sequentially to the previous one(s), assuming that these interventions are fully adhered to by all eligible patients with ischaemic stroke or transient ischaemic attack (TIA) in Scotland. Untreated recurrent stroke risks are derived from UK population-based stroke registry data.

A major risk factor for recurring vascular events or death is therefore non-adherence to medication, but only limited data are available on patient adherence to medication intended to prevent recurrent stroke. However, there is no reason to believe that stroke patients should demonstrate better adherence than in other chronic conditions. In fact, the reverse is more plausible, given that stroke often causes memory impairment, which is known to cause adherence problems. In a study of over 3,000 patients in Germany, Hamann et al. [10] reported that 84% were still taking aspirin at one-year post stroke, 77% oral anticoagulants, but only 61% who were prescribed clopidogrel at discharge were still taking it one year later. Sappok et al. [11] also reported from a follow-up study one year after stroke and found that only 70% of patients were still taking cholesterol-reducing treatment. Data from the Netherlands revealed that by 1 year after ischaemic stroke, 22% of patients who had been taking oral anticoagulation had stopped, half of whom did so "for non-medical reasons" such as perceived adverse effects, patient request etc. [12]. Thus, the available data on stroke patients suggest that adherence is often sub-optimal, and that many patients are consequently at a significantly increased risk of a further stroke and/or cardiovascular event.

### Fractionating Adherence

Adherence is the end result of a complex set of perceptions, attitudes, cognitive abilities, intentions and behaviours. It has proved useful to distinguish between deliberate non-adherence (intentional) and non-deliberate non-adherence (non-intentional) [1]. The aim of this pilot project is to improve intentional adherence by addressing beliefs that act as a barrier to adherence and to reduce non-intentional non-adherence by developing plans to help reduce forgetting.

#### Intentional non-adherence

Our theoretical framework is based around Leventhal's self-regulation theory [13]. This theory posits that patients have a common-sense model of their illness in terms of beliefs regarding how long a condition will last, whether it is acute or chronic, what sort of treatments will help, etc. Superimposed upon this framework of illness beliefs are beliefs about treatment, particularly the perceived necessity of medication versus concerns about possible harmful effects of medication [14]. Our recently completed study [15] on determinants of adherence in stroke patients supported self-regulation theory: it identified that stroke patients' concerns about their medication (e.g. dependence, toxicity, too many tablets) were key determinants of poor adherence. We therefore aim to elicit and attempt to modify erroneous beliefs about medication and stroke in this pilot randomised trial of a brief intervention.

#### Non-intentional non-adherence

Many patients forget to take their medication as directed. Our previous study on adherence in stroke patients established that cognitive impairment (as measured by the Mini Mental State Examination (MMSE)) was significantly associated with poor adherence [15]. An impressive body of evidence has accumulated showing that brief and easy-to-complete implementation intentions interventions are effective at reducing forgetting and in improving medication adherence [16]. These involve patients writing down **exactly when and where **they will take their medication, using the format of an if-then plan ("If it is time X in place Y and I am doing Z, then I will take my pill dose"). The evidence clearly indicates that if-then planning makes people highly sensitive to the cues that they have written down, and means that they can act swiftly and effortlessly as soon as these cues are encountered, thus environmentally cued habits are established. Implementation intentions remove the burden of having to think about and remember when to act by using environmental cues to trigger the desired behaviour. The load on prospective memory is reduced as habitual responses are established (e.g. first cup of tea at breakfast in the kitchen cues taking morning medication). In a recent example, Brown et al. [17] showed that a simple if-then plan in epilepsy patients resulted in intervention participants showing improved adherence relative to controls on all three outcomes: doses taken in total (93.4% vs. 79.1%), days that correct dose was taken (88.7% vs. 65.3%), and doses taken on schedule (78.8% vs. 55.3%), all p < .01. Importantly, participants with the greatest degree of cognitive impairment benefited most from the intervention [17].

### Framework

We have developed our intervention using the new Medical Research Council (MRC) Guidance on developing and evaluating complex interventions [18]. We have completed the development work of the MRC framework and have identified the evidence base, utilised an appropriate theoretical model of adherence (self-regulation), and identified process variables that relate to both intentional and non-intentional adherence. We now wish to embark on feasibility/piloting where we will test a brief intervention, in terms of recruitment, retention, acceptability and efficacy, and use the results of the pilot to inform the sample sizes required for a larger, more definitive trial. The intervention has two components, tackling both intentional and non-intentional non-adherence, and each component has a strong, supportive evidence base.

We recently found that 30% of stroke patients reported sub-optimal medication adherence at interview. Approximately one third of the variance in self-reported poor adherence was predicted by the following four variables: (1) younger age, (2) greater cognitive impairment, (3) lower perceptions of medication benefits, and particularly, (4) greater specific concerns about medications (toxicity, side effects etc.) [15]. Our qualitative interview findings confirmed the questionnaire results by showing that (a) medication concerns were key determinants of medication taking behaviour and also (b) the establishment of a habitual routine for medication taking was seen as vital. The findings from that study justify the evaluation of a pilot intervention trial aimed at targeting both intentional and non-intentional components, with the goal of improving medication adherence.

### Aims

To pilot the feasibility of a brief intervention in stroke patients exhibiting sub-optimal adherence with the aim of:

(a) establishing a better medication taking routine using an implementation intentions intervention

(b) eliciting and modifying any emergent erroneous beliefs regarding the patient's medication and their stroke.

We will test whether medication routines and beliefs are changeable, and if the results are promising, this will pave the way for a larger randomised controlled trial (RCT) to determine whether adherence is improved, physiological risk is changed (e.g. via reduction in blood pressure), and rate of recurrent vascular events is reduced.

### Research questions

(a) Is the brief intervention feasible, understandable and acceptable (e.g. regarding uptake/attrition)?

(b) Does the intervention improve adherence?

(c) Is improvement in adherence mediated by (i) changes in illness and medication beliefs and/or (ii) reduced forgetting?

(d) What effect size is observed to inform the power calculation for a larger, more definitive study?

## Methods/Design

### Recruitment

In order to maximise recruitment and the representativeness of the sample, we shall attempt to recruit and obtain consent from consecutive patients who are discharged from the Edinburgh Western General Hospital stroke units and clinics and who are prescribed secondary antihypertensive medication. Our previous experience suggests that this approach will significantly improve trial recruitment. Currently approximately 300 in-patients and 400 out-patients are discharged per year, with over 60% prescribed antihypertensive medication. We will screen 400 first-time stroke (ischaemic and haemorrhagic) or Transient Ischaemic Attack (TIA) patients, and expect a 75% response rate (300). We plan to include both stroke and TIA patients since both groups of patients are treated in a similar way with secondary prevention drugs and are likely to have similar issues with respect to non-adherence [9]. We have decided to focus on the early months following stroke in order to maximise the likelihood of preventing a further stroke. Poor adherence will have a much greater effect on stroke risk in the first few months because the risk of stroke (and thus the absolute risk reductions associated with drug treatment) is highest at this stage [19]. Furthermore, following Petrie et al. [20], we believe that the efficacy of the intervention will be enhanced by eliciting and correcting dysfunctional stroke and medication beliefs soon after preventative drugs are started. However, we have to strike a careful balance between early intervention, and also allowing enough time for participants to demonstrate variance in adherence, so that we can specifically target those showing sub-optimal adherence, thus our decision to assess adherence at 3 months post stroke or TIA. Consenting participants will therefore be contacted by post 3 months after their event and asked to complete the Medication Adherence Self Report Scale (MARS) [21] and return it in a stamped addressed envelope. In this mailing, we will also ask participants to complete the Brief Illness Perceptions Questionnaire [22] and the Beliefs about Medication Questionnaire [23]. This will allow us to economically test the robustness and replicability of the association we previously observed between specific medication concerns and self-reported adherence in a new large sample of TIA/stroke patients. We will then invite all those reporting sub-optimal adherence on the MARS (score of 24 or less) to participate. Based on our earlier study we conservatively estimate that approximately 30% will report some degree of poor adherence, and of those, up to 30% may then decline to participate. We therefore estimate that if we invite 90 patients, 60 will agree to participate. They will then be randomly allocated to brief intervention or treatment as usual (TAU). We are allowing for a further 10% attrition rate during the trial, however, one of the main purposes of this pilot is to obtain data on uptake, acceptability and attrition.

#### Inclusion criteria

We aim to be as inclusive as possible and recruit all patients who had their first ischaemic or haemorrhagic stroke or TIA 3 months earlier and were discharged from the ward or clinic on any secondary preventative medication and are living at home.

#### Exclusion criteria

We will only exclude people who are not on anti-hypertensive medication 3 months after their stroke/TIA, or whose degree of aphasia (Frenchay screen <13/20) or MMSE <23 makes completion of the study measures not feasible. Those who report already using Dosette boxes to improve their adherence, or are not responsible for taking their own medication, will also be excluded.

### Design

**Figure 1:**
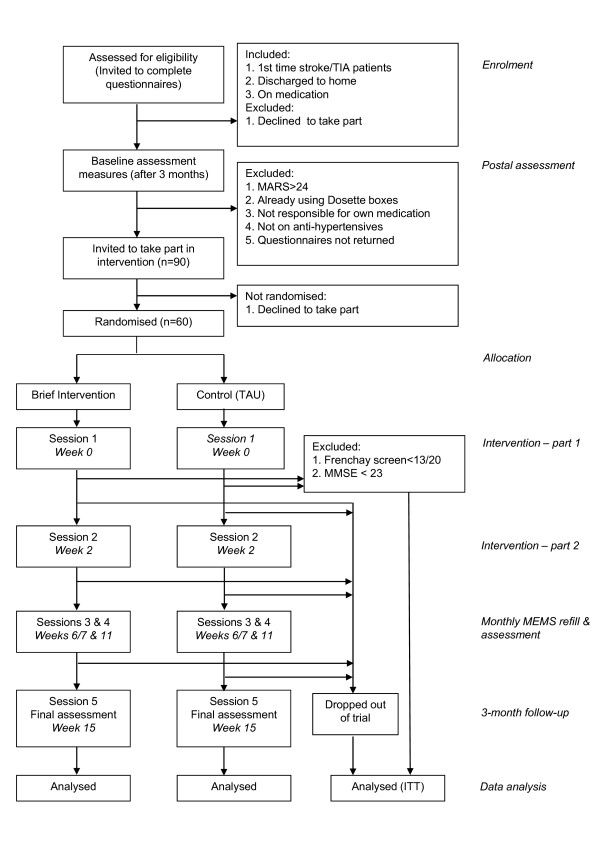
**CONSORT flowchart of trial design**.

A pilot RCT with patients allocated to intervention versus treatment as usual (TAU). Web-based randomisation at the patient level into intervention or TAU will be provided by the Edinburgh Clinical Trials Unit, using minimisation with a random element to ensure that the two trial arms are not significantly different on three key variables: age, number of pills taken per day and baseline MARS adherence. A CONSORT flowchart of the trial design is shown in Figure 1.

### Setting

A single-centre trial at a large teaching hospital in Scotland.

### Ethical Approval

Ethical approval has been granted by Lothian NHS Board, South East Scotland Research Ethics Committee (REC ref. no. 09/S1102/36).

### Measuring Adherence

There is no agreed "gold standard" when measuring adherence [21]. Our previous study clearly established that assay of urinary aspirin levels in stroke patients lacked sensitivity and was unhelpful [15]. Garber et al. [24] showed that appropriately framed self-report questionnaires show good concordance with electronic cap monitors and blood and urine measurement. The MARS attempts to reduce social desirability effects by framing questions so to make non-adherent responses socially acceptable. The MARS has high internal and test-retest reliability, and has been shown to predict clinical outcome (blood pressure within range [21]). We used the MARS as our primary outcome measure in our previous study, establishing that stroke patients found it easy to use and understand, and we demonstrated that MARS scores were not correlated with a social desirability measure but were prone to ceiling effects [15]. We will therefore use the MARS as a viable and economic screen for patients demonstrating sub-optimal adherence, but will then use MEMS (Medication Event Monitoring System, MEMS^® ^Aardex Ltd, Switzerland) pill containers which electronically record openings as our primary outcome measure in the evaluation of this pilot RCT.

### Intervention

Two brief sessions, two weeks apart with a trained Research Fellow, lasting approximately 30-45 minutes each. Participants will be given the choice of having home visits or coming into a local hospital-based Clinical Research Facility.

***Session 1 ***will focus on helping each patient draw up a specific plan, so as to establish a better medication-taking routine using an implementation intentions approach. Patients will be helped to complete an individualised worksheet plan for each scheduled daily dose of antihypertensive medication, following Brown et al. [17]. The participant and Research Fellow will both keep a copy of the plan. Baseline blood pressure readings will also be taken during Session 1.

***Session 2***. The effectiveness of the implementation intentions plan and any barriers/difficulties in following the plan will be reviewed in session 2, with individually-tailored coping strategies/plans developed collaboratively, following the methods outlined by Sniehotta et al. [25]. This session will also focus on eliciting and, if appropriate, challenging patients' beliefs regarding their medication, e.g. beliefs regarding toxicity, dependence, fears regarding medications interacting harmfully etc., using the participants' responses on the BIPQ and the BMQ as a basis. The aim here will be to correct any misperceptions and provide evidence so that participants' medication **necessity **beliefs come to outweigh their medication **concerns **beliefs. Previous work has demonstrated that better adherence results when medication necessity beliefs outweigh concerns [14]. Modification of erroneous beliefs about stroke will be based on the model of Petrie et al. [20] who elicited and modified patients' dysfunctional beliefs regarding their recent myocardial infarction. This resulted in faster return to work and lower angina symptoms at 3 months. If the Research Fellow is unable to answer any specific questions regarding the patient's stroke or medication, then immediately following the interview, the RA will email the query to one of the stroke consultant experts on the research team, and the RA will then telephone the patient with the information within 7 days of the interview. At the end of session 2, the Research Fellow will fill each participant's MEMS medication bottle with the following month's supply of antihypertensive medication. (We propose using the patients' existing supply of antihypertensive medication. A check will be taken at session 1, and if supplies are running low, participants will be asked to obtain their repeat prescription in advance of session 2). For each of the next three months (Sessions 3-5), the Research Fellow will repeat this process, and also take an electronic reading from the MEMS cap, downloading the data on to a laptop PC for later analysis. At the first of these follow-up visits (Session 3), participants will again complete the Brief Illness Perceptions Questionnaire (BIPQ) [22] and the Beliefs about Medications Questionnaire (BMQ) in order to test whether the intervention has resulted in changes to stroke and/or medication beliefs [23]. At the final visit, at 3-month follow-up (Session 5), the outcome measures will be administered.

### Control condition

Participants in the control group will receive the same number of Research Fellow visits and will complete the same questionnaires at the same timepoints as the intervention group. MEMS readings and BP recordings will also be taken, as detailed in the intervention arm. During the first 2 sessions, the Research Fellow will also engage control group participants in non-medication related conversation, e.g. how they are feeling, how they are spending their time etc. in an attempt to provide some control for non-specific effects of attention/social contact.

In both conditions, all interviews will be timed and digitally audio-recorded and transcribed for supervision feedback and check on treatment fidelity.

### Primary outcomes

Medication adherence will be recorded using MEMS (Medication Event Monitoring System, MEMS^® ^Aardex Ltd, Switzerland) pill containers which electronically record openings. Following Brown et al. [17], and in line with previous studies using this method, we shall use the following main outcomes, counting each opening as a presumptive dose: (a) percentage of doses taken (versus doses prescribed), (b) percentage of days on which the correct number of doses was taken, and (c) percentage of doses taken on schedule. Again, following Brown et al. [17], we designate doses as having been taken on schedule if the MEMS bottle was opened within a 3-hour (plus or minus) time window for each dose. The electronic monitoring caps can be connected to a personal computer that reads the data from the pill caps' microprocessors and generates a printout of every pill bottle opening over an extended time period (in this case, the preceding month). Because patients will usually be on a variety of medications, we will target antihypertensive medication for MEMS measurement, particularly as there is clear evidence regarding poorly treated blood pressure significantly increasing the risk of future vascular events, and as stated in the introduction, it is estimated that only 30-50% of patients regularly take their antihypertensive drugs as prescribed [2]. If patients are taking more than one antihypertensive, we shall target the drug that is taken most frequently. MEMS have been successfully used in a variety of medication adherence interventions e.g. Brown et al. [17]. However, as with most methods in this area, MEMS measurement is not immune from the Hawthorne effect, i.e. medication-taking behaviour is often improved in the short-term as a direct consequence of it being measured [26]. We shall therefore use MEMS containers to record medication taking in both intervention and control arms for 3 months. We predict in the control arm that adherence will gradually drop off over the 3 months (as the Hawthorne effect fades), whereas there will be an increase or no change over the 3 months in the intervention arm. A recent three-month RCT aimed at improving adherence to medication in HIV-affected individuals also used this MEMS evaluation of outcome, and in the intention to treat analysis showed no change in the TAU arm, but a significant improvement in the psychological intervention arm, with a controlled effect size of 1.0 [27]. We acknowledge the limitation in this pilot that the Research Fellow will not be blind to treatment arm; however, as our primary outcome is MEMS automated recording of days per month that the correct dose of hypertensive was taken, the potential for bias is significantly reduced.

Secondary outcomes will include (a) MARS self-reported adherence of all secondary preventative medication, (b) systolic and diastolic blood pressure.

### Analysis

MEMS data will be analysed using an intention to treat protocol in a repeated-measures mixed-design (2 groups * 3 time points). One of the primary aims of this pilot study is to determine the effect size achieved by this pilot to inform the sample size calculation for a larger, more definitive multi-centre study. (We acknowledge that the effect size observed may differ from what one would see in a large trial, however, the effect size sought in the large trial should be within the 95% CI of the estimate derived from this pilot. The pilot will thus give a useful measure of the variability of the primary outcome measures). Using G-Power [28] for this pilot, we calculate that with 2 arms of 30 participants, focusing on the treatment group by time interaction term, we should be able to detect an effect size of 0.2, with a power of 0.80 and alpha set at 0.05 (i.e. a 0.2 of a standard deviation improvement in the brief intervention arm in number of days per month that the correct antihypertensive medication is taken, relative to the TAU arm).

### Control variables

At Session 1, following Trewby et al. [29] and our previous study, all participants will be asked to complete a baseline measure of their perception of the benefit (0-100%) provided by their current stroke prevention medication using a simplified graphical presentation card. Participants will also complete the Mini-Mental State Examination (MMSE), as we previously demonstrated that younger age, lower MMSE and low perceived benefit of medication were all related to poor adherence [15]. Finally, we will determine whether these, and other baseline characteristics (e.g. prior use of reminder packaging and partner involvement in reminding to take medication), are related to treatment outcome.

### Evaluation

The effects of the intervention will be evaluated in all participants via measurement of the primary and secondary outcome variables listed above. The Research Fellow will also take baseline and 3-month follow-up blood pressure measurements using an OMRON M10-IT BP monitor following a standardised protocol (mean of 3 recordings). The blood pressure results will be fed back to the participants and their GPs in both intervention and TAU arms, together with simple information regarding ideal values. (NB we do not expect significant change in these physiological variables in this pilot, but these may change - albeit over a longer time period - in a larger trial and we wish to assess feasibility and patient acceptability). On completion, patients in the intervention arm only will also take part in a brief semi-structured interview to assess their views regarding the intervention. They will also be asked to complete Likert-type scales assessing (a) ease of understanding, (b) acceptability and (c) perceived benefit for each of the intervention components, namely, 1) medication routine planning worksheet, 2) discussion/information regarding medication, 3) discussion/information regarding stroke, and 4) blood pressure measurement. Additional file 1: Table S1 details all patient contacts and assessments at each time point.

#### Process evaluation

We will test if any change in adherence is mediated by changes in medication beliefs (Beliefs about Medications Questionnaire - BMQ) and/or illness beliefs (Brief Illness Perceptions Questionnaire).

### Timetable

This is a 31-month project. The Research Fellow will spend months 1-3 learning techniques for eliciting and modifying patients' dysfunctional beliefs regarding their stroke and their medication. Patients will be invited to participate from month 3 onwards. It is anticipated that the running of the trial will take 24 months (months 4-27). Postal recruitment will be used to collect baseline data on the MARS, BMQ and BIPQ self-report measures, and to identify participants who are suitable for the intervention. Each participant recruited into the intervention will participate for roughly four months, thus we envisage 6*4 month blocks, with approximately 10 patients being run concurrently in any one of these blocks. They will each require 5 face-to-face contacts (choice of home visit or meeting in the local Clinical Research Facility) over the participation period. Session 1 is when the remaining baseline measures will be completed and the implementation worksheet plan drawn up. Session 2 is two weeks later when the plan is reviewed and elicitation and modification of illness and medication beliefs will be conducted, and the MEMS containers filled for the following month. Sessions 3 and 4 are brief monthly meetings to take a MEMS cap reading and refill the MEMS bottle. BMQ and BIPQ scores will also be collected at Session 3. The final outcome measures will be taken at Session 5. Months 28-31 will be spent analysing the data and in preparing the final report and papers for conference presentation and publication.

## Discussion

Improving adherence to appropriate prescriptions of existing efficacious treatments may well represent the best investment for improving self-management of long-term medical conditions [1]. This work has the potential to improve adherence, treatment efficacy and reduce health service waste. A health economic evaluation would be central to any subsequent large-scale trial. This pilot trial is a logical development from our recent study of determinants of medication adherence in stroke patients [15], and is novel in that it is targeting both intentional and non-intentional non-adherence via belief modification and implementation intentions respectively. The intervention is based on self-regulation theory and is brief, practicable and capable of being delivered by trained non-specialist health workers in an NHS setting. In a recent critical evaluation of adherence interventions [21], 6 consistent weaknesses in the field were identified: 1) narrow focus for intervention, in particular a failure to consider both intentional and non intentional non-adherence, 2) "one size fits all" approach i.e. not patient-centred, 3) failure to specify the content of the intervention, 4) "black box" evaluation, 5) lack of theoretical framework and 6) little or no process evaluation. Our proposed study addresses each of these weaknesses in that we are tackling intentional and non-intentional non-adherence, are using a patient-centred approach by eliciting individualised concerns, are describing the nature and content of the intervention, thus allowing for replication, are using Leventhal's self-regulatory model as our theoretical framework and are testing process by assessing whether change in adherence is attributable to changes in illness and/or medication beliefs and/or reduced forgetting. This work has the potential to significantly improve the efficacy of a broad range of treatments in the NHS. If the results of the pilot are promising, a larger more definitive study in stroke patients would be planned and similar evaluations in other chronic conditions should also be considered.

## Competing interests

The authors declare that they have no competing interests.

## Authors' contributions

All the authors were directly involved in the discussions that led to the joint writing of this study protocol. All authors read and approved the final version of the study protocol.

## Pre-publication history

The pre-publication history for this paper can be accessed here:

http://www.biomedcentral.com/1471-2377/10/15/prepub

## Supplementary Material

Additional file 1 **Table S1: Details and schedule of IAMSS assessment procedures**. The table shows all patient contacts and details of the assessments taken at each time point. Click here for file
