# Supplementary material for: Improving adherence to medication in stroke survivors (IAMSS): a randomised controlled trial: study protocol
Source: BMC Neurol. 2010 Feb 24;10:15. doi: 10.1186/1471-2377-10-15 (PMC2838838; doi:10.1186/1471-2377-10-15)
Supplement: Additional file 1 — Table S1: Details and schedule of IAMSS assessment procedures. The table shows all patient contacts and details of the assessments taken at each time point. [file 1471-2377-10-15-S1.DOC]

Table S1: Details and schedule of IAMSS assessment procedures

|  | **Postal contacts** | | **Face-to-face contacts** | | | | |
| --- | --- | --- | --- | --- | --- | --- | --- |
| **Measure** | **1^st^ letter  (3 months after stroke/TIA)**  **Baseline measures** | **2^nd^ letter  (invited to take part in intervention)** | **Session 1 (intervention session 1)**  **(week 0)** | **Session 2 (intervention session 2)**  **(week 2)** | **Session 3**  **(refill pill bottles)**  **(week 6/7)** | **Session 4**  **(refill pill bottles)**  **(week 11)** | **Session 5  (final assessment)**  **(week 15/6)** |
| Check of exclusion criteria (e.g. Dosette box usage, BP medication) | X | - | - | - | - | - | - |
| Medicine Adherence Self-report Scale (MARS) [21] | X | - | - | - | - | - | X |
| Brief Illness Perception Questionnaire (BIPQ) [22] | X | - | - | - | X | - | X |
| Beliefs about Medication Questionnaire (BMQ) [23] | X | - | - | - | X | - | X |
| Age, daily medication regime | - | X | - | - | - | - | - |
| Frenchay Screen test | - | - | X | - | - | - | - |
| Mini Mental State Exam (MMSE) | - | - | X | - | - | - | - |
| Blood Pressure (Average of 3 readings) | - | - | X | - | - | - | X |
| Perception of benefit of medication (Trewby et al., 2002 [29]) | - | - | X | - | - | - | - |
| Medication Event Monitoring System (MEMS®) readings | - | - | - | - | X | X | X |
| Semi-structured interview on views of intervention | - | - | - | - | - | - | X |
| Likert Scales on a) ease of understanding, b) acceptability and c) perceived benefits of intervention components | - | - | - | - | - | - | X |
